# Apatite saturation revisited: new model formulations and applications to igneous rocks

**DOI:** 10.1007/s00410-026-02300-5

**Published:** 2026-02-19

**Authors:** Benjamin Z. Klein, Othmar Müntener, Jack Gillespie, Felix Marxer

**Affiliations:** 1 Institute of Earth Sciences, University of Lausanne, Lausanne, Switzerland; 2 Institute of Earth System Sciences, Leibniz University Hannover, Hannover, Germany

**Keywords:** Apatite, Accessory mineral saturation, Magmatic differentiation, Thermometry

## Abstract

Apatite is the primary phosphate mineral in the Earth’s crust and is present in a range of intrusive and extrusive igneous rocks, typically at minor to trace quantities. Similarly, apatite is a stable phase in many equilibrium crystallization experiments conducted with phosphorus-bearing starting compositions. We leverage this experimental stability to produce a large compilation of apatite-saturated liquid compositions, supplemented by additional apatite trace element and volatile partitioning and explicit apatite solubility experiments, as well as analyses of natural rhyolitic glasses. Using this compilation, we calibrate two new, independent models: for apatite saturation temperature as a function of melt P_2_O_5_ and SiO_2_ contents and aluminum saturation index (ASI; molar Al/(2Ca + Na + K)); and for melt P_2_O_5_ contents at apatite saturation as a function of temperature, melt SiO_2_ contents and ASI. The first model reproduces apatite saturation temperatures with an accuracy of ~ 32 °C, significantly outperforming existing apatite saturation models as well as recent zircon saturation thermometers. The second model reproduces melt P_2_O_5_ contents at apatite saturation by better than a factor of two across four orders of magnitude. Our new calibrations show that, within uncertainty, apatite stability does not differ regardless of the specific volatile species present (H_2_O, F or Cl) or on the quantity of H_2_O dissolved in the melt. Further, we find that apatite stability is not sensitive to pressures at least in the range of 1 atm to 2 GPa. This model accurately describes the saturation of apatite in a wide range of liquids, including metaluminous liquids, moderately alkaline liquids, and most peraluminous liquids. Experimental and natural peraluminous liquids with unusually high P_2_O_5_ contents that are not well described by our model contain CaO:P_2_O_5_ ratios below apatite stoichiometry (‘perphosphorous melts’), indicating that apatite saturation in these liquids is at minimum jointly controlled by CaO and P_2_O_5_ contents.

**Supplementary Information:**

The online version contains supplementary material available at 10.1007/s00410-026-02300-5.

## Introduction

Calcium phosphate apatites (Ca_5_(PO_4_)_3_(F,Cl,OH), hereafter collectively referred to as apatite) are the primary host of phosphorus in the Earth’s crust and are present in a range of intrusive and extrusive igneous rocks, typically as accessory phases (Webster and Piccoli 2015). Due to its common occurrence and ability to incorporate a range of elements in trace quantities, apatite has become a widely studied mineral in magmatic systems. The trace amounts of radioactive elements such as U, Th, Sm, and Lu contained within apatite have led to its use as a versatile mineral chronometer, with wide-spread use in fission track and U-Th-Sm/He thermochronology (Gleadow et al. 2002; Chew and Spikings 2015), in addition to geochronology applications using the U–Pb, Sm–Nd, and Lu–Hf systems (Simpson et al. 2021; Gillespie et al. 2022). Further, apatite volatile contents have been used to infer the evolution of volatiles in terrestrial magmas (e.g., Stock et al. 2016; Humphreys et al. 2021; Keller et al. 2023), and for materials from the Moon and Mars (Filiberto and Treiman 2009; McCubbin et al. 2011; Gross et al. 2013; Boyce et al. 2014; McCubbin and Jones 2015). Recent studies have also highlighted the potential for apatite to constrain the behavior of sulfur in magmatic systems (Streck and Dilles 1998; Parat and Holtz 2004; Economos et al. 2017), as well as to use the speciation of sulfur in apatite as an oxybarometer (Konecke et al. 2017, 2019; Brounce et al. 2019).

In mafic and intermediate systems, phosphorus is generally strongly incompatible in silicate phases, and therefore apatite plays the dominant role in controlling the magmatic evolution of phosphorus: phosphorus contents continuously increase in crystallizing melts prior to apatite saturation and decrease with continuing crystallization following apatite saturation (e.g., Hoskin et al. 2000; Lee and Bachmann 2014). Due to this systematic behavior, beginning in the 1980s and 1990s experimental studies aimed to develop models for apatite saturation as a function of melt temperature, phosphorus content, and melt composition (Watson 1979, 1980; Watson and Capobianco 1981; Green and Watson 1982; Harrison and Watson 1984; Bea et al. 1992; Pichavant et al. 1992; Wolf and London 1994). This paralleled the development of saturation models for several other common accessory phases (zircon, monazite and rutile).

While other accessory phases, particularly zircon, have seen additional experimental studies and refined saturation models since this early work (Boehnke et al. 2013; Gervasoni et al. 2016; Marxer and Ulmer 2019; Crisp and Berry 2022; 2024), apatite saturation has received comparatively little attention (c.f., Tollari et al. 2006) despite recognized limitations in the application of existing apatite saturation models (Yakymchuk and Acosta-Vigil 2019). By contrast to zirconium which is typically only included in experimental starting compositions in studies designed to examine an aspect of zircon crystallization, phosphorus is included in the starting materials of most experimental studies and is typically analyzed in experimental glasses. As a result, there is a large body of published apatite-saturated experiments that can be used to constrain apatite solubility. Here we review the existing models for apatite and phosphate saturation and then present a compilation of experimental liquids in equilibrium with apatite, which we use to both evaluate the existing models for apatite saturation and to develop a new model.

## Existing apatite saturation models

The first apatite saturation model, published by Harrison and Watson (1984), was calibrated using a combination of new experimental data and published data from a series of earlier experiments examining apatite stability in different melt compositions (Watson 1979, 1980; Watson and Capobianco 1981; Green and Watson 1982). These earlier studies were conducted with both metaluminous and peralkaline silicate melt compositions and included both experiments where new apatite crystallized from silicate starting compositions (Green and Watson 1982), and apatite dissolution experiments, wherein a silicate glass not initially saturated in apatite dissolves apatite until reaching equilibrium. Apatite saturation was imposed in dissolution experiments by combining silicate starting compositions with large quantities of apatite, either as the experimental capsule (Watson 1979), as finely ground apatite crystals (Watson 1980; Watson and Capobianco 1981) or as a single large, polished apatite grain surrounded by silicate glass (Harrison and Watson 1984). In most of these earlier experiments glass analyses were reported to be homogeneous, without evidence for diffusive boundary layers surrounding apatite grains. In contrast, the Harrison and Watson (1984) experiments were conducted to determine phosphorus diffusivity, and thus the equilibrium phosphorus concentration in the quenched glass at apatite saturation was taken as the peak phosphorus concentration at the interface between the apatite grain and silicate glass. Using this collection of experiments, Harrison and Watson (1984) derived the following model for apatite saturation (HW84):

1 $$\begin{array}{c}\mathrm{ln}{D}_{{\mathrm{P}}_{2}{{\mathrm{O}}}_{5}}^\frac{apatite}{melt}=\frac{8400+\left({w}_{{\mathrm{Si}}{\mathrm{O}}_{2}}-0.5\right)2.64\times 1{0}^{4}}{T(K)}-\left[3.1+12.4\left({w}_{{\mathrm{Si}}{\mathrm{O}}_{2}}-0.5\right)\right]\end{array}$$

where *T(K)* is the temperature in Kelvin, ${w}_{{\mathrm{Si}}{\mathrm{O}}_{2}}$ is the weight fraction SiO_2_ in the melt (denoted that way here to avoid confusion with weight percent: e.g., a melt with 75 wt. % SiO_2_ has a ${w}_{{\mathrm{Si}}{\mathrm{O}}_{2}}$ equal to 0.75), and ${D}_{{\mathrm{P}}_{2}{{\mathrm{O}}}_{5}}^\frac{apatite}{melt}$ represents the ratio of phosphorus content in apatite (typically taken as 42 wt.% P_2_O_5_) and in the melt. The original publication asserts that this model appears to be valid for melts containing between 45 and 75 wt. % SiO_2_ and 0–10 wt. % H_2_O and for temperatures between 850 and 1500 °C and is unlikely to be strongly pressure sensitive at crustal pressures.

The HW84 solubility model predicts very low apatite solubilities in evolved melts at temperatures near the granite minimum: a melt with 75 wt. % SiO_2_ at 750 °C in equilibrium with apatite is predicted to contain only 0.01 wt. % P_2_O_5_. This is in apparent contradiction with the existence of peraluminous granites that contain up to weight percent amounts of P_2_O_5_ (Bea et al. 1992; Bucholz 2022) without comparably high CaO contents, precluding significant accumulation apatite, and similarly with the peraluminous Macusani rhyolites, which contain glass with > 0.5 wt. % P_2_O_5_ but are apatite-free (e.g., Pichavant et al 1988). This observation motivated multiple subsequent studies that aimed to characterize the solubility of apatite in felsic, peraluminous hydrous melts (Bea et al. 1992; Pichavant et al. 1992; Wolf and London 1994). The study conducted by Pichavant and co-workers (1992) included both dissolution and crystallization experiments conducted at temperatures between 777 and 1000 °C. Dissolution experiments were conducted by mixing a haplogranitic (i.e., Ca, P free) silicate starting mix with finely crushed apatite and andalusite, while crystallization experiments were conducted on a Ca- and P-bearing granitic glass starting composition, again with crushed andalusite added to guarantee peraluminous melt compositions. Only experiments that produced melts with homogeneous phosphorus concentrations were used to calculate apatite solubility. Based on these experiments, Pichavant and co-authors (1992) asserted that apatite solubility is dependent on the aluminum saturation index (ASI; molar Al/(2Ca + Na + K) – note that in all studies referenced here ASI is calculated without accounting for phosphorus content) in addition to temperature and melt SiO_2_ contents, and proposed a modification to the HW84 apatite solubility model (PMR92):

2a $$\begin{array}{c}{P}_{2}{O}_{5}^{melt}={P}_{2}{O}_{5}^{HW}+{P}_{2}{O}_{5}^{Per};\end{array}$$

2b $$\begin{array}{c}{P}_{2}{O}_{5}^{Per}=\left(ASI-1\right)\mathrm{exp}\left(-\frac{5900\left(965\right)}{T(K)}-3.22\left(1.67\right){w}_{Si{O}_{2}}+9.31\left(0.77\right)\right)\end{array}$$

Here, ${P}_{2}{O}_{5}^{HW}$ is the phosphorus solubility calculated according to HW84 (Eq. 1), and ${P}_{2}{O}_{5}^{Per}$ is a correction for peraluminous melt compositions, intended to be applied when ASI > 1.

Bea and co-authors (1992) similarly observed that HW84 significantly underpredicted phosphorus contents when applied to a compilation of peraluminous granite bulk rock compositions. In this study, previously published pelite melting experiments that produced peraluminous liquid compositions (Holtz and Johannes 1991) were used to calibrate an alternate correction to the HW84 model that accounts for phosphorus behavior in peraluminous liquids (BFC92; note that the published model is calibrated unconventionally with temperature in degrees Celsius and for consistency with the other calibrations we have rewritten the calibration here with temperature in Kelvin):

3 $$\begin{array}{c}{P}_{2}{O}_{5}^{melt}= {P}_{2}{O}_{5}^{HW}\times \mathrm{exp}\left(\frac{6429\left(ASI-1\right)}{T\left(K\right)-273.15}\right)\end{array}$$

A final investigation of the effect of ASI on apatite solubility was conducted by Wolf and London (1994). In this study, similar to the previously discussed work by Pichavant and co-authors (1992), apatite solubility was evaluated by mixing a near-haplogranitic melt composition with apatite and activated amorphous Al_2_O_3_ which served as a source of excess aluminum. Addition of three different apatite components were evaluated: isolated coarse grained apatite; abundant finely crushed apatite; and abundant coarse grained apatite forming a nearly apatite grain-supported matrix. Experiments with coarse, isolated apatite grains were found to not reach equilibrium even with experimental durations of 15 weeks at 750 °C and moreover, the phosphorus concentration at apatite grain boundaries was found to vary with run duration. Experiments with abundant apatite grains, either finely crushed or coarse grained, produced melts with homogeneous phosphorus contents, and were used to calibrate a simple relationship between apatite solubility and ASI for melts with ASI ≥ 1.1 (WL94):

4 $$\begin{array}{c}{P}_{2}{O}_{5}^{melt}=-3.4+3.1ASI\end{array}$$

As this simple relationship does not account for the variation in apatite solubility as a function of temperature or melt SiO_2_ contents, it is only appropriate for melts with similar compositions as those used in these experiments, and at temperatures near 750 °C.

Finally, a more recent study by Tollari and co-workers (2006) revisited the topic of phosphorus solubility in silicate melts. This study presented a series of 1-atmosphere experiments conducted on anhydrous synthetic starting compositions that contained 5–10 wt. % P_2_O_5_ and were halogen free. As a result, apatite was not stable, and the experimental liquids instead crystallized whitlockite ((Ca,Mg,Fe)_3_(PO_4_)_2_). These results were combined with previously published data from apatite saturation experiments to develop a general model for phosphate solubility calibrated over a wide range of temperatures and melt compositions including peraluminous compositions (TTB06):

5 $$\begin{array}{c}{M}_{{P}_{2}{O}_{5} }=\mathrm{exp}\left(T(K)\left(\frac{-0.8579}{139-{M}_{Si{O}_{2}}}+0.0165\right)-\frac{10}{3}\mathrm{ln}\left({M}_{CaO}\right)\right)\end{array}$$

where *M*_*i*_ signifies the mole percent of component *i* in the liquid.

## Experimental compilation

A total of 783 liquid compositions coexisting with apatite were compiled from 79 publications (Table 1; complete compilation available in Supplemental Table S1). Of these experiments, 118 are dissolution-type experiments compiled from some of the publications used to calibrate existing apatite saturation models (Watson 1980; Watson and Green 1981; Wolf and London 1994), while the full melt compositions were not published in three of the original dissolution-type experimental studies (Green and Watson 1982; Harrison and Watson 1984; Pichavant et al. 1992) and thus these experiments could not be included in the compilation. 133 additional experiments were compiled from studies evaluating the partitioning of halogens, trace elements or sulfur between apatite and silicate melts (Parat and Holtz 2004, 2005; Mathez and Webster 2005; Prowatke and Klemme 2006; Webster et al. 2009; Doherty et al. 2014; Li and Hermann 2015, 2017; McCubbin et al. 2015; McCubbin and Ustunisik 2018; Ji and Dygert 2024). Many of these experiments were conducted with initially apatite undersaturated compositions mixed with apatite seed crystals, and thus in terms of major element systematics are apatite dissolution experiments. The remaining partitioning experiments were conducted with starting compositions with elevated phosphorus contents to ensure early apatite crystallization. The remaining 532 experiments were compiled from experimental studies that produced apatite saturated melts (hereafter referred to as crystallization/melting or C/M experiments). In nearly all these studies, melts were included in the compilation when the presence of apatite was explicitly noted, either in a table of experimental results or in the text. As apatite stability was rarely the focus of these studies and apatite is typically present in small amounts (often less than 1 wt. %) in experimental charges, a small number of additional experiments were included when apatite stability could reasonably be inferred. Two criteria were applied to identify these experiments: 1) when apatite was reported in the same publication in a higher temperature experiment conducted at the same pressure with the same starting composition, bulk H_2_O, and *f*O_2_; and 2) when the melt P_2_O_5_ contents decrease between the higher temperature, apatite saturated experiment and the lower temperature experiment. Additionally, a small number of experiments were excluded due to described evidence for disequilibrium (e.g., skeletal apatite), or when melt compositions had unusually high analytical uncertainties. These exceptional included and excluded experiments are explicitly noted in Supplementary Table 1.

**Table 1:** List of publications included in experimental data compilation

References	Experiment type	Starting composition type
Andújar et al. (2016)	C/M	Natural
Andújar et al. (2023)	C/M	Natural
Andújar et al. (2025)	C/M	Natural
Blatter et al. (2017)	C/M	Natural
Blatter et al. (2023)	C/M	Natural
Bogaerts et al. (2006)	C/M	Natural
Castro et al. (2013)	C/M	Natural
Chang and Audétat (2023)	C/M	Synthetic
Clarke et al. (2023)*	Dissolution	Synthetic
Dann et al. (2001)	C/M	Synthetic
Doherty et al. (2014)	Partitioning	Natural
Erdmann et al. (2015)	C/M	Natural
Erdmann and Koepke (2016a)	C/M	Natural
Erdmann and Koepke (2016b)	C/M	Natural
First et al. (2021)	C/M	Natural
Foley et al. (2022)*	C/M	Synthetic
Foley et al. (2025)*	C/M	Synthetic
France et al. (2010)*	C/M	Natural
Frascerra et al. (2024)	C/M	Natural
Green and Adam (2002)	C/M^1^	Synthetic
Grove et al. (1997)	C/M	Natural
Helz (1976)	C/M	Natural
Hermann and Spandler (2008)*	C/M	Synthetic
Holtz and Johannes (1991)	C/M	Natural
Iacovino et al. (2016)	C/M	Natural
Iveson et al. (2017)	C/M	Natural
Ji and Dygert (2024)	Partitioning^2^	Synthetic
Kawamoto (1996)	C/M	Natural
Koepke et al. (2018)	C/M	Synthetic
Li and Hermann (2015)*	Partitioning	Synthetic
Li and Hermann (2017)*	Partitioning	Synthetic
Lloyd et al. (1985)*	C/M	Synthetic
Mahood and Baker (1986)*	C/M	Natural
Mandler et al. (2014)	C/M	Natural
Marxer and Ulmer (2019)	C/M	Natural
Marxer et al. (2022)	C/M	Synthetic
Marxer et al. (2023)	C/M	Synthetic
Mathez and Webster (2005)	Partitioning	Synthetic
McCubbin et al. (2015)*	Partitioning	Synthetic
McCubbin and Ustunisik (2018)*	Partitioning	Synthetic
Minitti and Rutherford (2000)	C/M	Synthetic
Moussallam et al. (2013)	C/M	Natural
Mutch et al. (2016)*	C/M	Natural
Nandedkar et al. (2014)	C/M	Synthetic
Nekvasil et al. (2004)	C/M	Natural
Nekvasil et al. (2009)	C/M	Synthetic
Parat et al. (2008)	C/M	Natural
Parat and Holtz (2004)*	Partitioning	Synthetic
Parat and Holtz (2005)*	Partitioning	Synthetic
Pichavant (2022)*	C/M	Natural
Pichavant et al. (1992)*	Dissolution	Synthetic
Pilet et al. (2010)*	C/M	Synthetic
Pineda et al. (2021)	C/M	Natural
Prowatke and Klemme (2006)*	Partitioning	Synthetic
Qian and Hermann (2013)	C/M	Synthetic
Riker et al. (2015)*	C/M	Synthetic
Rondet et al. (2019)	C/M	Natural
Sauvalle et al. (2024)	C/M	Natural
Scoates et al. (2006)*	C/M	Natural
Singletary and Grove (2008)	C/M	Synthetic
Sisson et al. (2005)	C/M	Natural
Sisson and Kelemen (2018)	C/M	Natural
Sisson and Grove (1993)	C/M	Natural
Stechern et al. (2024)	C/M	Natural
Tollari et al. (2008)	C/M^1^	Synthetic
Ulmer et al. (2018)*	C/M	Synthetic
Vander Auwera et al. (1998)	C/M	Natural
Vukadinovic and Edgar (1993)*	C/M	Synthetic
Wang et al. (2019)	C/M	Natural
Wang et al. (2024)	C/M	Synthetic
Watson (1980)*	Dissolution^3^	Natural
Watson and Green (1981)	Dissolution	Natural
Weber and Castro (2017)*	C/M	Natural
Webster et al. (2009)	Partitioning	Natural
Whitaker et al. (2007)	C/M	Synthetic
Wolf and London (1994)	Dissolution	Synthetic
Wolff et al. (2013)	C/M	Natural
Xiong et al. (2005)	C/M	Natural
Xiong et al. (2009)	C/M	Synthetic

^*^References for which all experimental data were excluded from the calibration dataset due to criteria discussed in the text

^1^Crystallization experiments conducted with starting compositions containing excess phosphorus to induce early apatite saturation

^2^This study investigated the partitioning of trace elements between apatite and silicate melts. It was conducted similar to C/M experiments, in that it was conducted with an initially apatite-free starting material containing elevated P_2_O_5_ contents to ensure early apatite saturation

^3^Contains both dissolution experiments and starting compositions with excess phosphorus

Approximately 80% of these experiments used natural materials as starting compositions and thus are assumed to contain both fluorine and chlorine, while the remaining experiments were conducted with synthetic starting materials that are assumed to be halogen-free unless those components were added to the starting material. Nearly all experimental starting compositions were hydrous, although a small number of anhydrous experiments with halogen-bearing starting compositions are also included. H_2_O contents of experimental glasses were compiled when reported in the original publications. When not reported, melt H_2_O contents were calculated, either by mass balance when melts were fluid-undersaturated, or with alphaMelts (v2.3.1; Smith and Asimow 2005; Ghiorso and Gualda 2015; Antoshechkina 2024), when melts were fluid saturated. These methods have been shown previously to reasonably reproduce directly measured melt H_2_O contents (e.g., Devine et al. 1995; Klein et al. 2023). In rare instances when the experimental documentation was insufficient to use either of these two methods, H_2_O contents were estimated as the deficit in the microprobe total.

This compilation of literature data was supplemented by new analyses of experiments from two publications (Nandedkar et al. 2014; Marxer and Ulmer 2019). The tonalite crystallization study by Marxer and Ulmer (2019) contained several apatite-bearing experiments, but phosphorus contents of the melts were not measured in the original publication. New analyses of these experimental melts were made by electron microprobe (EPMA) using the JEOL JXA-8530 HyperProbe at the University of Lausanne. The study by Nandedkar and co-authors (2014) consists of a series of fractional crystallization experiments that produced experimental charges with high melt fractions over a range of temperatures, including evolved, apatite-bearing melts. These high melt fractions made it possible to re-analyze these experiments by laser ablation inductively coupled plasma mass spectrometry (LA-ICP-MS) using an Element XR sector field ICP-MS at the University of Lausanne. These new analyses have significantly lower analytical uncertainties than the EPMA measurements published in the original publication, but the two methods agree within uncertainty. Complete analytical details for both sets of new analyses are provided in the Supplementary Materials.

**Fig. 1:**
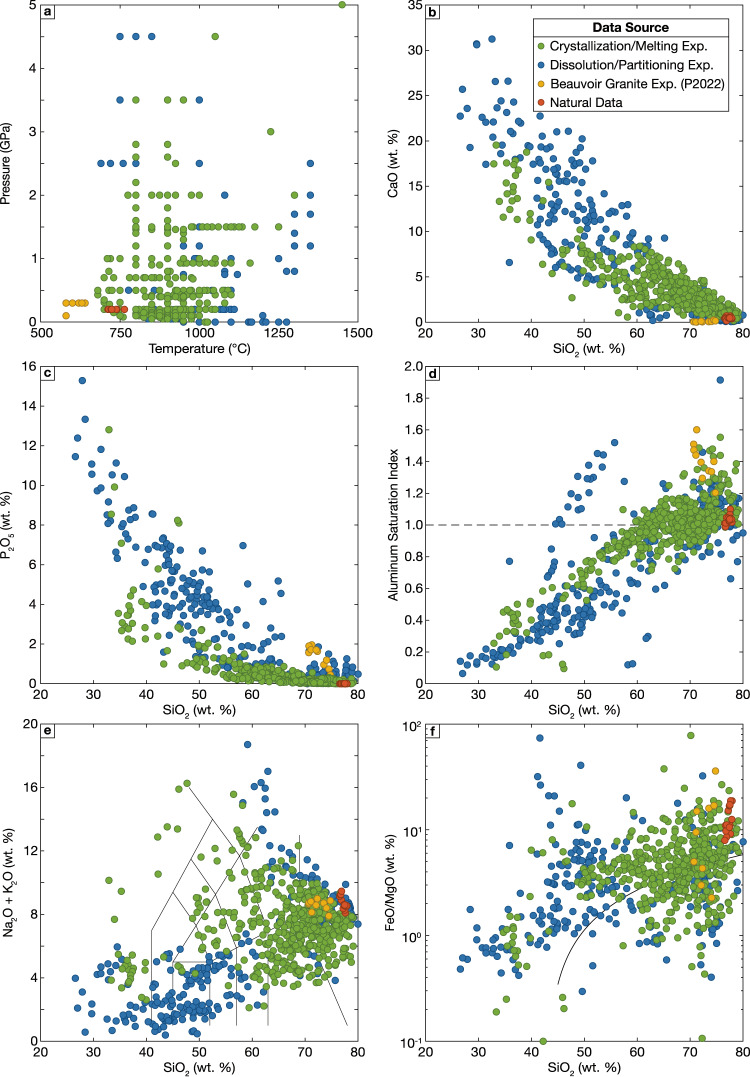
Summary of compiled experimental data. **a** Temperature and pressure conditions of compiled experiments. Natural rhyolite data arbitrarily plotted at 0.2 GPa. **b**, **c** Harker diagrams comparing CaO and P_2_O_5_ with SiO_2_ contents of experimental melts. **d** Aluminum saturation index (molar Al/(2Ca + Na + K)) compared to SiO_2_ of experimental liquids. **e** Total alkali-silica plot (volcanic rock classification fields after Le Maitre et al. 2005). **f** FeO/MgO (wt. %) compared to SiO_2_, with division between iron-enriched tholeiitic liquids and iron-depleted calc-alkaline liquids from Miyashiro (1974). In all plots, colors indicate experimental type (i.e., melting/crystallization or dissolution/partitioning), with natural data and experiments on strongly peraluminous Beauvoir Granite (P2022: Pichavant 2022) shown separately

Compiled experiments were conducted at pressures from 1 atm to 5 GPa and temperatures from 580 to 1450 °C (Fig. 1a), but the vast majority of experiments were conducted at pressures ≤ 2 GPa and temperatures between 700 and 1200 °C. The compositions of experimental apatite-saturated melts span a large range. C/M experimental melts (shown in green in Fig. 1) vary in composition from extremely silica-poor liquids enriched in calcium and phosphorus to typical rhyolites (Fig. 1b, c) but dominantly consist of intermediate and felsic compositions. The C/M melts cover a broad variation in alkaline, metaluminous and peraluminous compositions (Fig. 1d, e) and include recent experiments studying the strongly peraluminous, Li-, P- and F-rich Beauvoir Granite (Pichavant 2022), which are separately plotted in yellow. Tholeiitic (Fe-enrichment) and calc-alkaline (Fe-depletion) trends are both represented (Fig. 1f). The dissolution and partitioning experiments (shown in blue in Fig. 1) span a similar compositional range, but the dominant population is shifted to lower silica contents. Given this extremely broad compositional range, most of the remainder of the manuscript relies on a dataset filtered to exclude the most extreme compositions and focus on compositions broadly relevant to typical terrestrial magmas. The following criteria were used to produce this restricted dataset based on normalized anhydrous melt compositions: SiO_2_ ≥ 45 wt.%; Na_2_O ≤ 10 wt. %; K_2_O ≤ 10 wt. %; experimental pressure ≤ 2 GPa; and experimental temperatures between 680 and 1150 °C.

Finally, as noted earlier, existing models predict that evolved compositions near the fluid-saturated granite minimum should contain very low P_2_O_5_ contents (potentially ≤ 0.01 wt. %), at least for metaluminous melts. Experimental melt compositions are typically measured only by EPMA, and these low phosphorus contents are likely to be at or very close to the detection limit in typical EPMA measurements. Therefore, we supplemented the experimental compilation with analyses of phosphorus contents in natural apatite-bearing rhyolitic glasses by LA-ICP-MS from five publications (more examples in the literature were not found as phosphorus is typically not measured during routine LA-ICP-MS analyses). Compared to experiments, larger uncertainties exist in constraining the pre-eruptive storage temperatures of natural rhyolites; details of the natural data included in the compilation and the constraints on their magmatic temperatures are provided in Table 2.

**Table 2:** Natural rhyolite glasses included in compilation

Eruptive Unit	Glass Comp Ref	Temperature	Temperature Method	Temperature Ref
Peach Springs Tuff	Foley et al. (2020)	740 ± 25 °C	Zr-in-Titanite on titanite rims	Pamukcu et al. (2013)
Fish Canyon Tuff	Bachmann et al. (2002)^1^	712 ± 48 °C	Published average of multiple methods	Brückel et al. (2023)
Mesa Falls Tuff	Neukampf et al. (2019)	764 ± 26 °C	Ti-in-Zircon	Rivera et al. (2016)
Bishop Tuff	Gualda et al. (2022)^1^	714–738 ± 15 °C	Glass zircon saturation temperatures	Gualda et al. (2022)
Wolverine Creek Tuff	Szymanowski et al. (2015)	716 ± 14 °C	Ti-in-Zircon on zircon rims	Szymanowski et al. (2016)

^1^Fish Canyon and Bishop Tuffs both contain multiple glass analyses from distinct units. See Supplementary Table 1 for temperature estimates for individual units

**Fig. 2:**
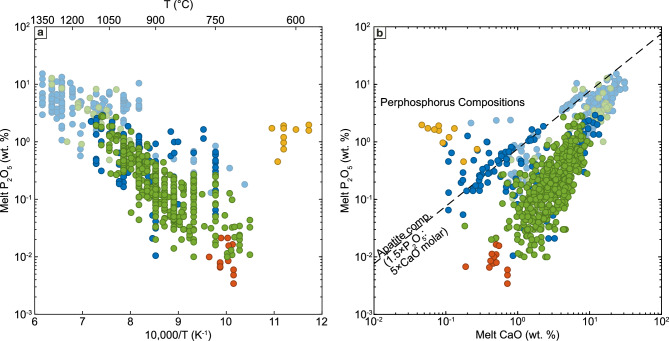
**a** Plot of compiled experimental data showing the broadly linear relationship between the log_10_ phosphorus contents of experimental melts and the inverse of experimental temperature. Note that the experiments on the Beauvoir Granite (Pichavant 2022) do not lie on the trend defined by the remainder of the data. **b** CaO and P_2_O_5_ contents of experiment melt compositions. Dashed line indicates normative apatite composition (5 mol CaO per 1.5 mol of P_2_O_5_). Compositions above this line are perphosphorus following the nomenclature of Bea et al. (1992). In both panels, symbols are as in Fig. 1, with experiments excluded based on pressure, temperature and compositional criteria described in the text shown with semi-transparent symbols

Most of the compiled data define a robust linear correlation between the inverse of experimental temperature and the logarithm of phosphorus contents (Fig. 2a). However, even after filtering as described above to remove the most extreme compositions, dissolution and partitioning experiments show significantly more scatter compared to the C/M experimental liquids, exhibiting more than an order of magnitude larger scatter in phosphorus contents at a given temperature. Much of this variability can be understood when comparing the CaO and P_2_O_5_ contents of the compiled liquids (Fig. 2b). This comparison shows three different populations, delimited by the CaO:P_2_O_5_ ratio of stoichiometric apatite (molar ratio of 5 CaO: 1.5 P_2_O_5_). The first and largest population is defined by excess CaO and consists of nearly all the C/M experiments, the natural rhyolite glass compositions, plus some dissolution-type glasses. For these compositions, apatite stability is expected to be dictated by the availability of phosphorus. The second population lies on the apatite stoichiometry line and consists of a large subset of the dissolution-type glasses. This trend indicates that both the CaO and the P_2_O_5_ in the melt are dominated by dissolving apatite (i.e., an apatite control line). Finally, a third, much smaller population has excess P_2_O_5_ relative to apatite, with compositions previously defined as ‘perphosphorus’ liquids following the nomenclature of Bea et al. (1992). This population consists of a small number of dissolution-type experiments and the C/M experiments conducted on the Beauvoir Granite, a strongly peraluminous, Li-, P- and F-rich granite that has a solidus below 600 °C (Pichavant 2022). In these liquids, the availability of CaO will be the limiting factor controlling apatite saturation. As the present study is focused on apatite solubility in typical silicate liquids (which are not perphosphorus), we subsequently use only the first population for evaluating apatite saturation models. However, we will address apatite solubility in perphosphorus liquids as well other types of silicate melts excluded from the calibration dataset later in the discussion.

## Evaluation of existing models for apatite saturation

All the apatite/phosphate saturation models except for WL94 predict that the phosphorus contents of apatite/phosphate saturated melts are a function of melt equilibration temperature in addition to melt composition. Thus, it is possible to algebraically rearrange each of these models to serve either as predictors of P_2_O_5_ contents or temperature of apatite saturation. While this rearrangement has significant limitations (see discussion in following section on new model development), it is currently common practice (e.g., Piccoli and Candela 1994; 2001). Therefore, we separately evaluate each of the models in both forms: as a predictor of melt phosphorus contents as a function of melt composition and temperature, or as a thermometer based on melt phosphorus contents and other melt compositional variables.

**Fig. 3:**
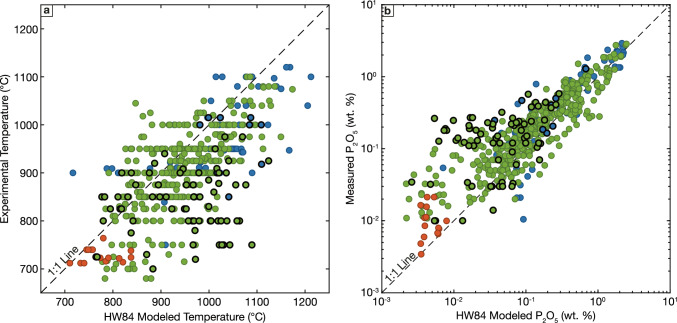
Evaluation of temperatures (**a**) and phosphorus contents (**b**) calculated using the model proposed by Harrison and Watson (1984) compared to experimental data. Only data that satisfy the filtering criteria discussed in the text are plotted. Symbology as in Fig. 1, with experiments with ASI > 1.1 highlighted with black outline

We begin by evaluating HW84, the most widely used model and the basis for two of the subsequent corrections for peraluminous liquids. Temperatures predicted by this model are positively correlated with experimental temperature but show significant scatter (Fig. 3a). HW84 modeled temperatures have a root mean squared error (RMSE) of 90 °C. Further, there is a significant bias in this scatter: the mean signed difference (MSD) of the dataset is +50 °C, indicating a persistent overprediction of experimental temperatures. As previous workers have noted (e.g., Bea et al. 1992; Pichavant et al. 1992; Yakymchuk and Acosta-Vigil 2019), peraluminous melt compositions are modeled poorly by HW84. Excluding these experiments and considering only crystallization/melting experimental data with ASI < 1.1 improves the RMSE of HW84 modeled temperatures to 75 °C and the MSD to +40 °C. Errors on HW84 modeled phosphorus contents exhibit a complementary behavior, where there is bias towards underestimating melt phosphorus contents (Fig. 3b). The scatter is particularly large at low temperatures, where HW84 underpredicts phosphorus contents by more than an order of magnitude for many experiments. Finally, we note that HW84 performs slightly better when considering only the natural rhyolitic melt compositions but still exhibits large errors and biased estimates: modeled temperatures have a RMSE of 61 °C and an MSD of 47 °C, while modeled melt P_2_O_5_ contents show a complimentary pattern, with underestimated values for nearly all samples. While the larger uncertainties on the temperature constraints for the natural samples may contribute to some of these errors, it is unlikely that the errors on these modeled values, and particularly the large average bias in the estimates, can be attributed to the temperature estimates alone.

**Fig. 4:**
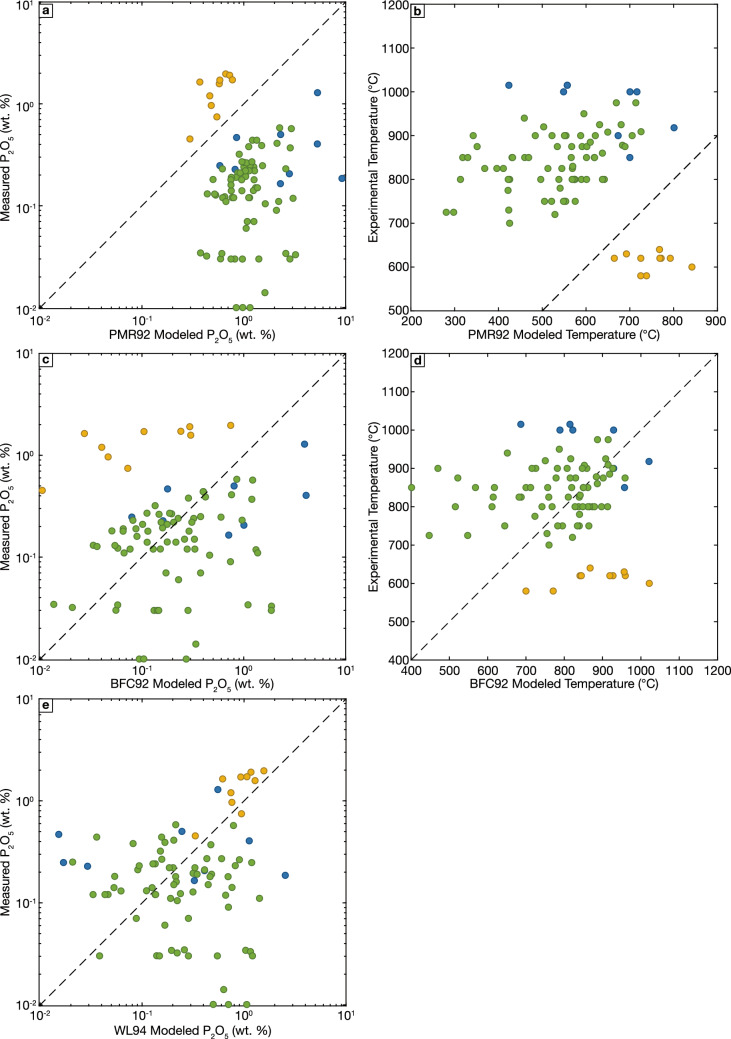
Evaluation of temperatures and phosphorus contents calculated using the three apatite saturation models developed for peraluminous liquids. Only data with ASI > 1.1 that also satisfy the filtering criteria discussed in the text are plotted. Note that temperatures calculated using PMR92 must be found numerically, while solving the BFC92 model (Eq. 3) for temperature yields a quadratic equation such that there are two valid temperatures solutions for every experiment. Plotted here are the higher temperature solutions; low temperature solutions typically range between ~ 0–200 °C. Colors as in Fig. 1

We next evaluate the performance of the phosphorus saturation models developed for peraluminous liquids, considering only experiments with ASI > 1.1 in Fig. 4, but also including the strongly peraluminous, perphosphorus Beauvoir Granite experimental liquids (Pichavant 2022; these liquids are excluded from Fig. 3 due to their low temperatures and perphosphorus compositions, but we note the HW84 models describe these low temperature, P_2_O_5_-rich liquids extremely poorly, overestimating experimental temperatures by > 600 °C and underestimating melt P_2_O_5_ by nearly three orders of magnitude.) Although both PMR92 and BFC92 can be used for any melt compositions with ASI > 1, we limit the evaluation to ASI > 1.1 as WL94 is only valid for these compositions, and the adjustments relative to HW84 will be comparatively minor using PMR92 and BFC92 for melts with 1 > ASI > 1.1. Using these data, we find that PMR92 significantly overcorrects HW84 for nearly every peraluminous experiment, resulting in significantly overpredicted melt phosphorus contents and underpredicted experimental temperatures (Fig. 4a, b). The only exceptions to this behavior are the Beauvoir Granite experimental liquids (Pichavant 2022), for which PMR92 moderately overpredicts experimental temperatures and underpredicts liquid P_2_O_5_ contents. Similar, but less extreme results are obtained using BFC92: model values show a moderate bias towards underestimating experimental temperature and overestimating melt phosphorus contents (Fig. 4c, d), although the bias is reduced compared to PMR92, and a portion of experimental compositions are accurately predicted using this correction. As with PMR92, the Beauvoir Granite experiments are anomalous, and the BFC92 correction is not sufficient to correct the significantly overpredicted temperatures and underpredicted phosphorus contents predicted by the unmodified HW84 model. Lastly, predicted melt phosphorus contents modeled using WL94 show reduced bias compared to other models, although the scatter in modeled P_2_O_5_ is still large: a large portion of predicted phosphorus contents are still incorrect by more than an order of magnitude. Of the four models considered, only the comparatively simple WL94 model accurately predicts the phosphorus contents in the Beauvoir Granite experiments. We speculate that this is a result of WL94 being calibrated on perphosphorus compositions broadly similar to the Beauvoir Granite experimental liquids. Collectively, these findings are broadly consistent with previous evaluation of these models using natural peraluminous melt inclusions and nanogranitoids (Yakymchuk and Acosta-Vigil 2019).

Finally, a comparison of experimental temperatures and equilibrium phosphorus contents with predicted values using the TTB06 model is presented in Supplementary Fig. 1. We find this model extrapolates poorly and, particularly for typical apatite-bearing evolved, silica-rich and calcium-poor compositions, produces unreasonable predictions (e.g., negative temperatures and > 100 wt. % P_2_O_5_ for all melts with ≤ 2 wt. % CaO). This poor extrapolation is evident in the original publication, where modeled phosphorus contents in phosphate-saturated melts appear to increase exponentially with decreasing CaO contents (Tollari et al. 2006; their Fig. 13b). As this model predicts geologically unreasonable phosphorus concentrations and physically impossible temperatures for many experiments, it will not be evaluated in detail here.

## Development of a new apatite saturation model

Given the poor performance of existing apatite saturation models as evaluated with our compiled experimental dataset, we used these data to develop a new model for apatite saturation. Our approach to model fitting differs slightly from previous apatite (and zircon) saturation models. Typically, these models are derived by multiple regression of inverse temperature plus one or more compositional variables (± pressure) to predict the experimental melt P_2_O_5_ contents. This approach has a clear thermodynamic basis (see discussions in e.g., Harrison and Watson 1984; Tollari et al. 2006; Sartori and Schmidt 2023). However, the resulting equation predicting the phosphorus contents of melt at apatite saturation is infrequently used when interrogating natural samples; instead, the equation is commonly rearranged by solving algebraically for temperature, producing an apatite saturation thermometer. This form is also increasingly used to evaluate the quality of model fits (c.f., Crisp and Berry 2022). However, this practice is not mathematically rigorous for two reasons. (1) The algebraic rearrangement ignores the treatment of errors in standard least squares regression, where all errors are attributed to the response variable. (2) In multiple regression, the coefficient for each predictor variable describes the effect of that predictor on the response variable while keeping all other predictors constant; these coefficients do not describe relationships within the set of predictors themselves. Therefore, multiple regression equations are not generally algebraically invertible without altering their statistical meaning. Here we take a different approach, wherein we develop two independent empirical models, one for calculating temperature, and one for calculating P_2_O_5_ contents.

Models were fit using only the data from experiments that satisfied the filtering criteria described above. During initial fitting, data from experiments published in three publications (Mutch et al. 2016; Weber and Castro 2017; Ulmer et al. 2018) were identified as consistently diverging from the systematics defined by the bulk of the data and were excluded. We do not speculate here on the cause of the divergent behavior in these three publications but note that many of these experiments contained melts with P_2_O_5_ contents near typical EPMA detection limits. Finally, six additional individual experiments were identified as outliers during model refinement and were also excluded (excluded experiments are noted in Supplementary Table 1). The resulting dataset consists of 463 experiments drawn from 55 publications, supplemented by analyses of 15 natural rhyolite glasses from 5 additional publications.

Based on existing apatite saturation models, we expected that the relationship between temperature and phosphorus contents would also be a function of one or more additional terms that describe melt chemistry and/or structure. We evaluated a range of variables for this purpose including (all variables calculated as weight % unless otherwise noted): SiO_2_, ASI (molar Al/(2Ca + Na + K), xMg (molar Mg/(Mg + Fe)), FeO^T^/MgO, total alkalis (Na_2_O + K_2_O), H_2_O, and NBO/T (the molar ratio of non-bridging oxygens to tetrahedrally coordinated cations), as well as variables used previously to describe melt structure in zircon saturation models: M (Watson and Harrison 1983), G (Gervasoni et al. 2016) and optical basicity (Crisp and Berry 2022). In addition, we tested the performance of model behavior using the thermodynamically based simplified apatite solubility product ([CaO]^5^[PO_2.5_]^3^) in place of melt phosphorus contents (c.f., Tollari et al. 2006; Sartori and Schmidt 2023). Combinations of individual terms, as well as interaction and quadratic terms were evaluated using least squares multiple regression. Thermometer models were developed using non-linear least squares, while linear least squares were used to model log_10_(P_2_O_5_) as a function of inverse temperature and melt composition to produce a model with log-normal residuals. Based on this process, we found that the best model performance was achieved using models that followed the form used by HW84, but with an additional term describing a linear relationship between log(P_2_O_5_) and ASI.

**Fig. 5:**
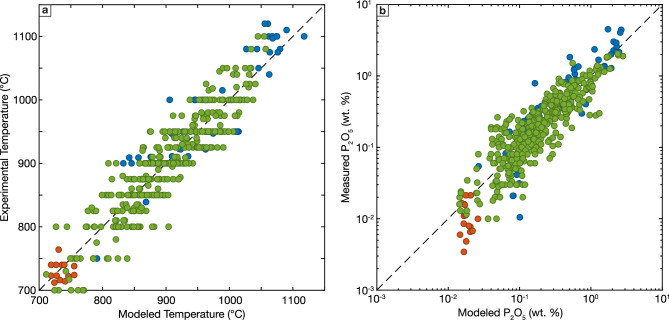
Evaluation of new apatite saturation models. **a** Comparison of experimental temperatures and temperatures modeled with apatite saturation thermometer (Eq. 6). **b** Comparison of measured and modeled melt P_2_O_5_ contents (wt. %; Eq. 7). Colors as in Fig. 1

Our proposed apatite saturation thermometer is:

6 $$\begin{array}{c}T\left(K\right)=\frac{{10}^{4}(6.71\left(0.60\right)-7.15\left(0.73\right){w}_{Si{O}_{2}})}{-{\mathrm{log}}_{10}\left({P}_{2}{O}_{5}\right)+50.15\left(4.47\right)-55.6{\left(5.6\right)w}_{Si{O}_{2}}+2.50(0.23)ASI}\end{array}$$

where parentheses give standard errors on model coefficients. This model reproduces experimental temperatures remarkably well (Fig. 5a): predicted temperatures have a RMSE of 32.4 °C, and a negligible MSD of -0.01 °C, indicating no bias in the predicted temperature data. Errors are consistent across the range of experimental temperatures and melt SiO_2_ contents. Model performance decreases slightly when considering only peraluminous liquids: the RMSE calculated for experiments with melt ASI ≥ 1.1 is 36.9 °C, while bias in modeled temperature for these experiments is still negligible (MSD = 0.3 °C), showing that the model can be successfully applied to both metaluminous and most peraluminous systems (but see later discussion about limitations in applying to peraluminous systems that are also perphosphorus).

Using the same functional form, but re-fit to directly model melt phosphorus contents at apatite saturation yields the following equation:

7 $$ \begin{array}{*{20}c} \begin{aligned} {\mathrm{log}}_{{10}} \left( {P_{2} O_{5} } \right) = & \frac{{10^{4} ( - 1.47\left( {0.22} \right) + 1.28(0.28)w_{{SiO_{2} }} )}}{{T(K)}} \\ & + 12.79\left( {1.65} \right) + 1.06\left( {0.12} \right)ASI \\ & - 14.06\left( {2.24} \right)w_{{SiO_{2} }} \\ \end{aligned} \\ \end{array} $$

This model successfully reproduces the range of phosphorus contents in experimental melts spanning nearly 4 orders of magnitude. The RMSE of the model is 0.25 log_10_ units, equivalent to a factor of ~ 1.8. It should be emphasized that by virtue of calibrating this model using linear least squares to reproduce log_10_(P_2_O_5_), the residuals are log-normal. This approach yields a model with errors that are proportional to the phosphorus contents, such that in linear space melt phosphorus contents are modeled more accurately in experiments with low phosphorus than in experiments with high phosphorus melts. The alternative approach of using non-linear least squares to directly model the (linear) melt P_2_O_5_ contents would produce a model with roughly constant errors, such that errors would be smaller for high phosphorus experiments, but modeled P_2_O_5_ in low temperature, phosphorus-poor liquids could be incorrect by more than an order of magnitude. We opted for this approach as we anticipate that our model will be applied dominantly to natural apatite-bearing intermediate to felsic magmas with comparatively low temperatures and phosphorus contents.

**Fig. 6:**
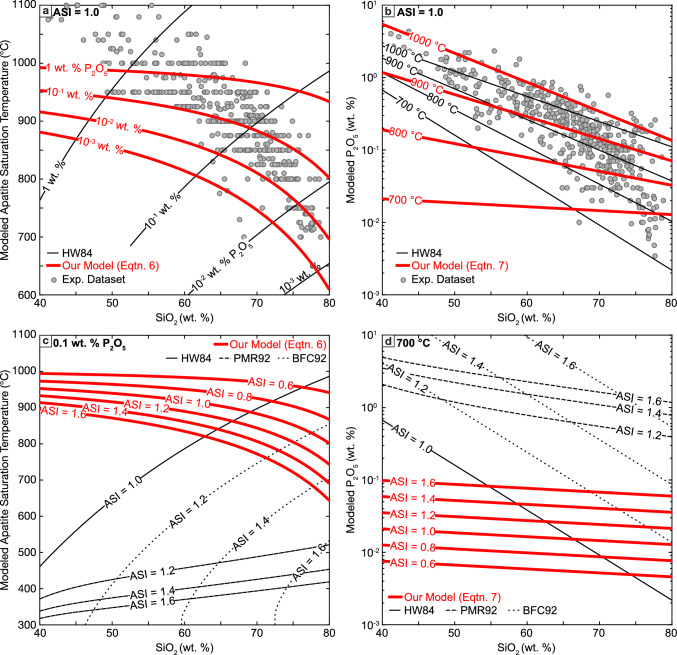
Comparison of new apatite saturation models to existing models. **a** Modeled apatite saturation temperatures as a function of melt SiO_2_ contents, comparing new saturation thermometer (red curves, Eq. 6) to temperatures calculated with HW84 (black curves). Curves show calculated saturation temperatures for constant melt P_2_O_5_ contents (labeled). **b** Modeled melt P_2_O_5_ contents as a function of melt SiO_2_ contents and at the labeled temperature, comparing new saturation model (red curves, Eq. 7) and HW84 (black curves). In **a** and **b** gray points show actual melt compositions and experimental temperatures of compiled apatite saturated experiments, and all red curves are calculated for ASI = 1. **c**, **d** Comparison of new apatite saturation models to modified peraluminous melt models. All curves calculated for a constant 0.1 wt. % P_2_O_5_. Red curves show new saturation models (Eq. 6 in panel c, Eq. 7 in panel d), dashed and dotted black curves show PMR92 and BFC92 models respectively. Solid black curve shows HW84 model (ASI independent) for context

Although Eqs. 6 and 7 share a similar form with HW84, they describe significantly different functional relationships between temperature and SiO_2_ and P_2_O_5_ contents (Fig. 6a, b). Both our models show a weaker dependence on SiO_2_ contents compared to HW84, and Eq. 6 finds a negative relationship between SiO_2_ and modeled apatite saturation temperature for a given melt P_2_O_5_ content, in contrast to the positive correlation predicted by HW84 (Fig. 6a). However, we note that the differences between the new models and HW84 are less pronounced when only considering the composition-temperature space sampled by the experimental dataset, represented by the gray points: Fig. 6a shows curves of apatite saturation temperature calculated for a given melt P_2_O_5_ contents as a function of melt SiO_2_ using both HW84 and our new model. These lines typically intersect near the center of the compiled dataset and diverge significantly only when extrapolated to conditions far outside of those expected for apatite saturation in natural magmas.

Figure 6 also illustrates the effect of changes to aluminum saturation index: Eq. 6 predicts that an increase in ASI from 0.8 to 1.2 shifts the apatite saturation temperature by ~ 100 °C in granitic liquids (Fig. 6c), while Eq. 7 finds that the same shift changes the melt P_2_O_5_ contents at apatite saturation by more than a factor of 2 (Fig. 6d). We also compare our new models with temperatures and pressures predicted by the ASI-sensitive PMR92 and BFC92 models in Figs. 6c and d. While the direction of the correction for increasing ASI is similar between the two existing models and our new models, the magnitude of the shifts predicted by PMR92 and BFC92 is much larger than for our new model. Further, we note that the PMR92 predicts an extremely non-linear, rapid response to increasing ASI above 1, a behavior not predicted by either our model or BFC92.

## Discussion

### Experimental equilibrium and accuracy of phosphorus measurements

Our approach of using compiled literature data is possible because phosphorus is commonly included in experimental starting compositions and measured during routine EPMA analyses of experimental glasses. However, only a very small subset of the crystallization/melting experimental studies used to calibrate the apatite saturation models presented here were conducted with a particular focus on phosphate stability or phosphorus behavior. Therefore, two concerns arise: (1) were phosphorus measurements during routine analyses consistently accurate enough to justify our approach, and (2) were the experiments in equilibrium with respect to apatite and phosphorus contents? The quality of phosphorus EPMA measurements is difficult to evaluate; it is a minor component in experiment melts and rarely the primary focus of the compiled experimental publications. Thus, even large errors in phosphorus measurements could go undetected in published datasets. Further, phosphorus contents in low temperature, evolved melts are typically near microprobe detection limits. Finally, analyses of melt phosphorus contents adjacent to apatite grains may be elevated due to secondary fluorescence. One factor that limits the potential impact of inaccuracies in phosphorus measurements is that, like previous models, our apatite saturation model is calibrated based on the logarithm of phosphorus contents. This makes it robust to modest errors in measured phosphorus. Further, as our compiled dataset incorporates data from numerous EPMA facilities and spans many years, if a large fraction of the phosphorus analyses were of poor quality or there were systematic biases between facilities, it is unlikely that the cumulative errors in the compiled dataset would have a coherent direction. That is, we would expect that if the phosphorus data were poor, the compiled data would appear to be both imprecise and inaccurate, resulting in poor model performance. Therefore, we argue that the performance of the thermometer calibration (Eq. 6) attests to the quality of the measurements, while the coherency between the modeled temperatures of high-silica experimental glasses and natural rhyolites, which have phosphorus contents measured by LA-ICP-MS with significantly lower detection limits, suggests that the data are reliable even in low phosphorus experimental glasses.

**Fig. 7:**
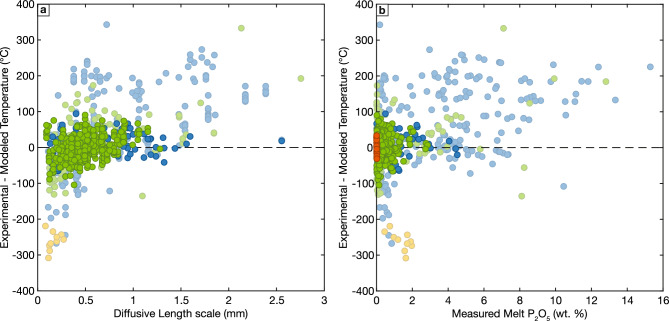
Comparison of apatite saturation thermometer residuals (experimental – modeled temperature) with **a** diffusive length scale calculated from experimental duration and temperature-dependent phosphorus diffusivity from Watson and co-workers (2015); and **b** melt P_2_O_5_ contents (wt. %). Colors as in Fig. 1, with semi-transparent symbols showing experimental data excluded from model calibration

Attainment of equilibrium in the compiled experiments is similarly indicated by the quality of the calibrated models. If a large fraction of the experiments did not reach equilibrium, there would be significantly more scatter in the calibration data, and again, coherency between the model predictions for experimental and natural melt compositions would be unlikely. While phosphorus has been shown to be a relatively slow diffusing element in silicate melts (Harrison and Watson 1984; Wolf and London 1994; Watson et al. 2015), slow phosphorus diffusivity appears to not hamper crystallization/melting type experiments. This is demonstrated in Fig. 7a where we show that the residuals of the modeled apatite saturation temperature (experimental – modeled temperature) is nearly invariant in the calibration dataset with respect to the diffusive length scale estimated from the experimental duration and temperature-dependent phosphorus diffusivity (Watson et al. 2015). If slow phosphorus diffusion in the liquid was a significant experimental problem, we would expect to see systematically increasing residuals in experiments with short diffusive length scales (corresponding to low temperatures and/or short durations). Although phosphorus diffusivity is also a function of melt composition and would be significantly slower in silica-rich melts (c.f., Wolf and London 1994; Harrison and Watson 1984), this lower diffusivity would only result in shorter calculated diffusive length scales of evolved melts, which already plot at the shortest diffusive length scale values in Fig. 7a.

The apparent minimal influence of phosphorus diffusion in the calibration dataset raises the question of why there is such a significant discrepancy between the calibration dataset and many of the excluded dissolution and partitioning experiments. These experiments would seem most sensitive to slow phosphorus diffusion as apatite dissolution inevitably produces phosphorus enriched boundary layers in the surrounding melt (c.f., Wolf and London 1994). However, the residuals calculated for these experiments, shown in semi-transparent blue symbols in Fig. 7a, do not consistently vary with diffusive length scale and instead are consistently large and generally positive regardless of diffusive length scale. This indicates that a different factor causes the large apparent differences between these experiments and the calibration dataset.

In detail, only a subset of the excluded publications presenting dissolution/partitioning experiments comprise the populations with large residuals shown in Fig. 7a. Some of these experiments were conducted on haplogranite starting compositions (Parat and Holtz 2004, 2005). These experiments plot on the apatite control line in Fig. 2b, suggesting that both CaO and P_2_O_5_ play important roles in apatite stability. Further, phosphorus has been shown to strongly interact with Fe in silicate melts (Toplis et al. 1994; Borisov et al. 2013), and the absence of Fe in these experiments likely also modifies the behavior of phosphorus in the melt. A second commonality shared by many of the excluded dissolution and partitioning experiments with large apatite saturation thermometer residuals are extremely high P_2_O_5_ contents. Indeed, the largest residuals are dominantly restricted to experiments containing melts with phosphorus contents greater than 5 wt. % (Fig. 7b). Numerous studies have shown that as phosphorus contents increase, it has an increasingly strong influence on melt structure (Ryerson and Hess 1980; Mysen et al. 1981; Toplis and Dingwell 1996) and should no longer be expected to behave as a Henrian component in the melt. Due to these effects, we recommend caution in applying our thermometer to the (rare) natural melts where phosphorus contents are greater than ~ 3 wt. %.

## The role of pressure and melt volatile contents

**Fig. 8:**
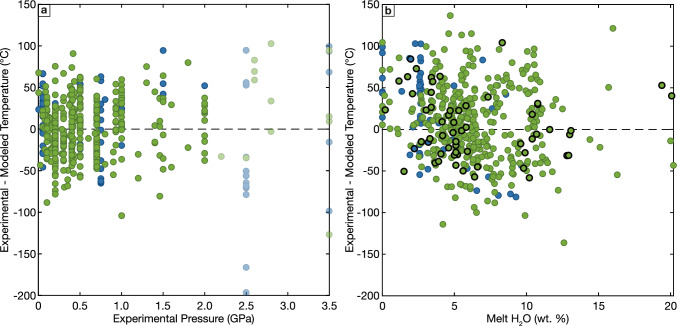
Evaluation of influence of additional terms on apatite saturation. **a** Comparison of apatite saturation thermometer residuals (experimental – modeled temperature) with experimental pressure. Semi-transparent symbols show experimental data excluded from model calibration due to high experimental pressure (> 2 GPa), but that otherwise would not have been excluded. **b** Comparison of apatite saturation thermometer with melt H_2_O contents (wt. %). Colors as in Fig. 1. Symbols outlined in black show experiments conducted with synthetic starting compositions that are halogen free, all other experiments conducted on either natural starting material assumed to contain both F and Cl or synthetic starting compositions that contained at least one of F or Cl

Our apatite saturation models and compiled data allow us to independently evaluate the sensitivity of apatite solubility to additional parameters not directly included in the models. Consistent with previous studies (Green and Adam 2002; Harrison and Watson 1984), comparing our model residuals to experimental pressure suggests that apatite saturation shows no pressure dependence up to 2 GPa. There are a small number of experiments that were excluded from our calibration dataset solely due to being conducted at higher pressures. Modeled apatite saturation temperatures for these experiments show relatively large residuals but do not exhibit a coherent trend with increasing pressure (Fig. 8a), suggesting that while the apatite saturation model performance deteriorates at high pressures, apatite saturation does not have a systematic pressure sensitivity at pressures up to ~ 3.5 GPa.

Similarly, apatite saturation thermometer residuals are not correlated with experimental melt H_2_O contents (Fig. 8b; note that for the numerous experiments conducted at H_2_O saturated conditions, melt H_2_O contents co-vary strongly with experimental pressure). Further, the model residuals are consistent between experiments conducted using synthetic (halogen free) starting materials shown with black outlines in Fig. 8b and experiments conducted on halogen-bearing natural starting materials. The apatite saturation model also performs comparably well for the small number of halogen-bearing, anhydrous experiments (experiments plotting on the y-axis in Fig. 8b). These results collectively indicate that, remarkably, if a suitable volatile species that can populate the column anion sites in apatite is present in the melt, apatite saturation is insensitive to the relative abundances of the individual volatile species. While the partitioning of these species between melt and apatite and the predominance of F- and OH-rich apatites in natural magmatic rocks (Mathez and Webster 2005; Webster and Piccoli 2015; Li and Costa 2020) both indicate that apatite stability must be sensitive to these species, the effect is apparently sufficiently small to not have a resolvable effect on apatite saturation given the available data.

## Extension of model to melt compositions outside of calibration set

While the apatite saturation models presented here were calibrated based on a large range of experimental melt compositions, there are additional, more unusual melt compositional spaces where the stability of apatite, or more generally of phosphates, is of interest. Here we evaluate the performance of our new apatite saturation models when extrapolating to three of these regimes: strongly peraluminous melts, strongly peralkaline and/or SiO_2_-poor liquids, and melts that stabilize phosphate minerals other than apatite.

### Strongly peraluminous liquids

**Fig. 9:**
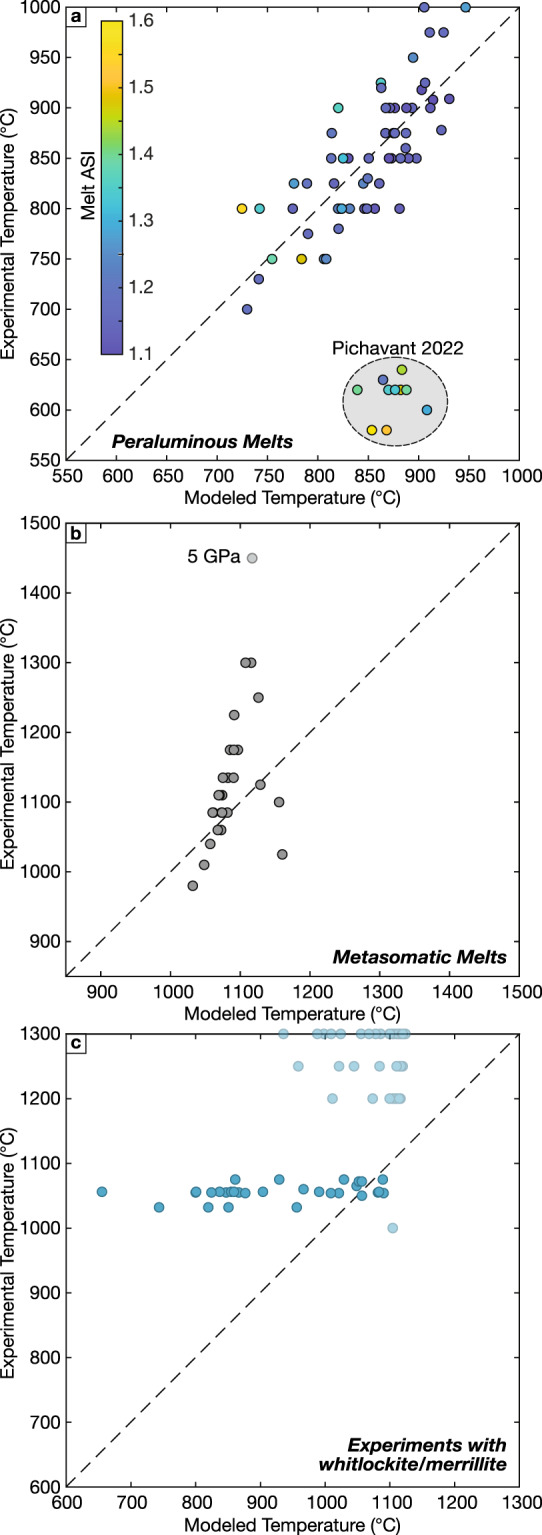
Evaluation of model performance when extrapolating beyond calibration dataset. **a** Evaluation of apatite saturation thermometer for moderately to strongly peraluminous (ASI ≥ 1.1) experiments. Experiments conducted on Beauvoir Granite from Pichavant (2022) diverge significantly from experimental calibration despite not having unusually high melt ASI. **b** Application of apatite saturation thermometer to melts in equilibrium with metasomatic assemblages that were excluded from model calibration due to exceptionally high alkali contents (Na_2_O or K_2_O ≥ 10 wt. %) and/or low SiO_2_ ≤ 45 wt. %. **c** Application of apatite saturation thermometer to anhydrous experiments that do not crystallize apatite, but rather a volatile free phosphate (typically whitlockite). Experimental data shown in panel c are from Tollari et al. (2006); Sha (2000); Vander Auwera et al. (1998); Toplis et al. (1994) and Rapp and Draper (2018)

The experimental calibration dataset contains many peraluminous melt compositions extending to ASI values above 1.5. As discussed previously, model performance is only slightly degraded for these peraluminous melts compared to the entire calibration dataset as expected given that ASI is one of the terms in both apatite saturation models. However, this performance does not extend to the recently published experimental data on the Beauvoir Granite (Pichavant 2022). The melts in these experiments are Li-, F- and P-rich, and are characterized by solidi below 600 °C. Modeled apatite saturation temperatures for these experiments are overestimated by 250–300 °C (Fig. 9a). Thus, despite the ability to successfully model most peraluminous melts, our apatite saturation models do not accurately model these unusual experimental compositions, and by extension, are unlikely to accurately describe natural P-rich peraluminous liquids (e.g., Bea et al. 1992; Bucholz 2022). However, as the model performs well with other liquids with comparable ASI, we speculate that the main factor responsible for suppressing apatite crystallization and generating high-P_2_O_5_ peraluminous liquids cannot be the peraluminous nature of the liquids. Instead, it seems that apatite stability in highly evolved compositions like the Beauvoir Granite are dominantly controlled by CaO contents. As described previously, these experiments all have molar CaO:P_2_O_5_ ratios less than apatite stoichiometry and are ‘perphorsphorous’ melts according to the terminology of Bea and co-workers (1992). In these perphosphorous compositions CaO is expected to behave as the limiting species, and as a result apatite saturation becomes comparatively insensitive to melt P_2_O_5_ contents. The Beauvoir Granite experiments published by Pichavant (2022) are unique in our calibration dataset in this regard and suggest that our apatite saturation models should not be applied to perphosphorous compositions. Given the limited experimental data on these systems, more experiments with varying melt compositions that span the gap between the Beauvoir Granite compositions and the bulk of the experimental database are needed to be able to model these unusual compositions.

### Alkaline liquids and metasomatized mantle melts

Determining the stability of apatite during mantle melting was the motivation for the earliest apatite saturation study (Watson 1980), and experimental research on apatite stability in metasomatic mantle melts is ongoing (c.f., Foley et al. 2025). These experiments primarily studied melts in equilibrium with ultramafic assemblages observed in metasomatic mantle veins which typically contain clinopyroxene + amphibole ± phlogopite ± apatite ± carbonates. We evaluate the ability of our apatite saturation model to reproduce the phosphorus systematics in the resulting alkaline melts in Fig. 9b, restricting this plot to experiments conducted on carbonate- (and CO_2_-) free metasomatic assemblages. These melts were all excluded from the calibration dataset due to either high experimental temperatures, very low melt SiO_2_ contents (< 45 wt. %, often much less), and/or highly elevated alkaline contents (Na_2_O or K_2_O > 10 wt. %). Perhaps unsurprisingly given that these melt compositions are far outside of the calibration dataset, model performance is poor in reproducing these experimental conditions. We do not evaluate the ability of our models to predict apatite saturation in carbonatitic liquids (c.f., Sartori and Schmidt 2023), but given the unacceptable performance for alkaline melts shown in Fig. 9b we are confident that model performance would deteriorate further when extrapolating to carbonatites. Based on these results, we emphasize that our apatite saturation models should not be used for varieties of melts that are poorly represented in our calibration dataset.

### Experiments that crystallize other phosphates

Finally, apatite is not the only stable phosphate phase observed in igneous systems. Evolved melts commonly crystallize the REE-phosphate monazite and rarely xenotime, while in anhydrous, halogen-poor experimental liquids whitlockite or merrillite instead crystallize (e.g., Sha 2000; Tollari et al. 2006). The stabilities of monazite and xenotime have seen considerable attention, and recent saturation models have been developed for both phases (e.g. Stepanov et al. 2012; Duc-Tin and Keppler 2015). These studies demonstrate that the stability of these phases is at least in large part dictated by the concentrations of REE and Y in the melt, and thus we do not consider these phases further here. By contrast, the stability of volatile-free phosphates including whitlockite and merrillite is poorly constrained. While these phases are rare in terrestrial magmas (c.f., Ionov et al. 2006), understanding their stability has importance for the study of meteorites, and lunar and Martian materials, where they are more common (e.g., Buchwald 1984; Jolliff et al. 2006). Further, a previous study suggested that apatite and these other, volatile-free phosphates can be modeled by a common ‘phosphate saturation’ model (Tollari et al. 2006). We test if this approach can be extended to our apatite saturation thermometer in Fig. 9c, where we plot the modeled ‘apatite’ saturation temperatures of experiments that crystallized either whitlockite or merrillite. Contrasting with the previous study by Tollari and co-workers (2006), our apatite saturation model performs poorly in predicting the phosphate saturation temperatures of these liquids, and significantly underpredicts the temperatures of most experiments containing melts in equilibrium with whitlockite or merrillite, including experiments that have melt composition and temperatures that fall within the range of our apatite calibration database.

## Application of new apatite saturation models to natural systems

All accessory phase saturation thermometers yield magmatic temperatures strictly only when applied to melt compositions in equilibrium with the accessory phase. The most straightforward application is therefore to analyze quenched glass in extrusive rocks, or bulk rock compositions of low crystallinity extrusive rocks. In these scenarios, saturation temperatures correspond to true magmatic temperatures (Hanchar and Watson 2003). A second readily interpreted scenario is the case of an intrusive rock that represents a liquid composition that has undergone only equilibrium (closed system) crystallization and when the modeled accessory phase is the liquidus phase. In this case the saturation temperature corresponds to the liquidus temperature (i.e., when the liquid reached accessory phase saturation). When applied to an intrusive rock that underwent equilibrium crystallization but where the accessory mineral was not the liquidus phase, saturation thermometers calculated from bulk compositions will tend to underestimate the actual temperature at which phase saturation occurs (Harrison et al. 2007). However, this dynamic is more complicated for apatite saturation models as it will be sensitive to the interplay between phosphorus and silica contents of fractionating melts. Finally, when applied to a wider range of intrusive rock compositions including granites with accumulated phases (either major or accessory), the significance of calculated saturation temperatures is more difficult to interpret: accumulated (and/or inherited) apatite will produce a bulk rock composition enriched in P_2_O_5_ contents relative to the liquid composition, raising calculated saturation temperatures (e.g., Miller et al. 2007; Siégel et al. 2018; Barnes et al. 2019). While accumulated major phases will modestly dilute the P_2_O_5_ contents, the more significant effect in many cases will be to lower the bulk rock SiO_2_ contents, resulting in elevated apatite saturation temperatures. Despite these nuances and challenges, saturation thermometers are widely used to interrogate the crystallization history of intrusive igneous rocks.

Given these different scenarios with potentially complex interpretations for apatite saturation temperatures, here we illustrate the utility of our new apatite saturation thermometer using three case studies: (1) Hekla Volcano in Iceland: an application to crystal-poor lavas where saturation temperature should correspond to eruption temperatures; (2) a compilation of whole rock data from the Cascades volcanic arc (compilation from Klein et al. 2023): a more complex application to a suite of extrusive rocks, some of which may not closely approximate liquid compositions; and (3) a diverse set of granitoids that were previously used to examine zircon saturation thermometry in intrusive rocks (Miller et al. 2003). These samples were previously characterized based on evidence for zircon inheritance; we use these samples to examine the coherency between zircon and apatite saturation systematics, and more generally to highlight some of the complications associated with interpreting saturation thermometers in intrusive systems.

### Case study #1 – Hekla

**Fig. 10:**
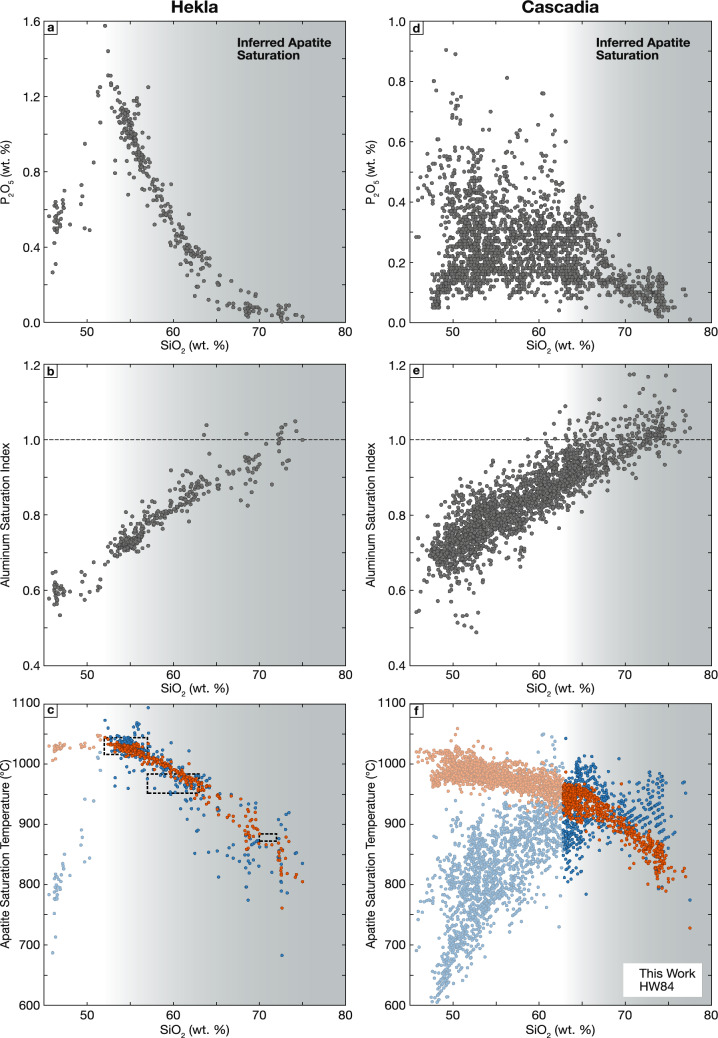
Evaluation of apatite saturation thermometry applied to natural extrusive rocks. **a**-**c** Application to whole rock and matrix glass data from Hekla volcano in Iceland (Baldridge et al. 1973; Jakobsson 1979; Óskarsson et al. 1982; Sigmarsson et al. 1992, 2022; Sverrisdottir 2007; Chekol et al. 2011; Savage et al. 2011; Larsen et al. 2019; Geist et al. 2021). Boxes with black dashed outline in panel c define temperature ranges calculated by Geist and co-workers (2021) using clinopyroxene-melt and zircon saturation thermometers. **d**-**f** Application to compiled whole rock data for Cascadia subduction zone (Klein et al. 2023). In all panels, gray field indicates region of inferred apatite saturation based on continuous decrease in P_2_O_5_ contents

Hekla is a volcano in Iceland that over the last ~ 1000 years has erupted a suite of crystal-poor lavas varying from basaltic andesite to rhyolite. This compositional variation appears to be dominantly generated by fractional crystallization, although the most differentiated compositions appear to not be strictly consanguineous with the less evolved products (e.g., Geist et al. 2021). The liquid line of descent defined by Hekla lavas illustrates the role of apatite saturation and subsequent fractionation, with increasing P_2_O_5_ contents in pre-historic basaltic lavas, followed by a peak in P_2_O_5_ contents of ~ 1.4 wt. % in basaltic andesites, and a continuous decrease in P_2_O_5_ contents with further differentiation through to rhyolitic compositions (Fig. 10a; Sigmarsson 2022). As Hekla lavas are crystal poor (crystallinity dominantly < 10%; Geist et al. 2021), their bulk compositions closely approximate liquid compositions, and apatite saturation temperatures should yield eruption temperatures for apatite saturated samples. Using our new apatite saturation thermometer, apatite saturation temperatures decrease from ~ 1050 °C in basaltic andesites down to ~ 850 °C in the most felsic rhyolite (Fig. 10c). These temperatures closely agree with previously calculated clinopyroxene-melt thermometry in basaltic andesites and andesites and with zircon saturation temperatures in rhyolites (Geist et al. 2021) and also are broadly in agreement with apatite saturation temperatures calculated using HW84, although HW84 temperatures show significantly more scatter. The two models diverge notably in the modeled (apparent) apatite saturation temperatures of basaltic melts that are assumed to be not apatite saturated, highlighting the divergence between the two models when misapplied to liquids not in equilibrium with apatite. This difference is discussed further in the following case study.

### Case study #2 – Cascadia

Figure 10d and e shows compiled whole rock data from the Cascadia volcanic arc used to calculate apatite saturation temperatures, which are shown in Fig. 10f. These data are undoubtedly reflective of multiple processes: distinct liquid lines of descent generated by fractionation of different parental melts, as well as varying amounts of crystal accumulation, magma mixing and crustal assimilation. Despite these complexities, if lavas are dominantly crystal-poor, apatite saturation thermometers should still closely approximate magmatic temperatures in apatite-bearing magmas. We conservatively infer that most Cascades lavas with > 65 wt.% SiO_2_ are apatite saturated based on the continuous decrease in P_2_O_5_ contents in Cascades dacites and rhyolites observed in Fig. 10d. Apatite saturation temperatures calculated for these felsic lavas define a relatively tightly bounded, continuously decreasing array with temperatures between 900 and 950 °C at 65 wt. % SiO_2_, falling to ~ 800–850 °C at 75 wt. % SiO_2_. These temperatures are consistent with experimental studies examining the production of Cascadia hydrous felsic melts (e.g., Sisson et al. 2005; Mandler et al. 2014).

In contrast to the Hekla example, apatite saturation temperatures calculated using HW84 diverge significantly from those calculated using our new thermometer. The greatest differences between the two thermometers are present in the temperatures calculated for basalts and basaltic andesites – liquids that are almost certainly not apatite saturated. These calculated temperatures theoretically should represent the temperature at which these liquids saturate in apatite. However, these conditions are extrapolated significantly beyond the calibration dataset (and at least in the case of HW84, the low calculated apatite saturation temperatures are almost certainly below the solidi of the basalt and basaltic-andesite liquids), and thus it is doubtful that they accurately predict the temperatures of eventual apatite saturation. More importantly, large differences are also present in the likely apatite saturated felsic compositions: in contrast to our thermometer, HW84 temperatures show significant scatter but do not define a clear trend of decreasing temperature with differentiation, resulting in dacite temperatures that are likely underestimated and rhyolite temperatures that are almost certainly overestimated. The systematic differences between the two thermometers likely have multiple causes. HW84 is more sensitive to changes in melt P_2_O_5_ than our thermometer – therefore it is possible that for some felsic samples, elevated temperatures in HW84 compared to our thermometer are reflective of accumulated apatite, evident in the increased P_2_O_5_ contents of some rhyolitic samples in Fig. 10d. Further, Cascadia liquids show a progressive increase in ASI with differentiation; the impact of this shift on apatite solubility is described by our model but not by HW84.

### Case study #3 – Granite compilation of Miller et al. 2003

**Fig. 11:**
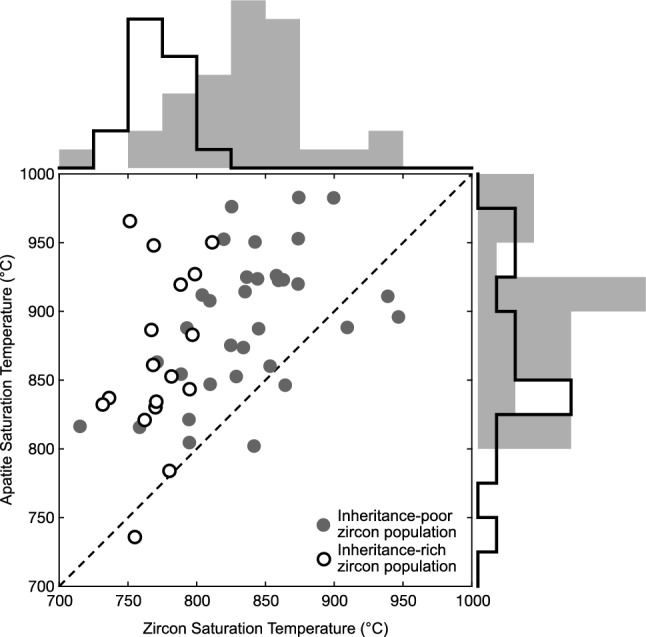
Comparison of calculated zircon and apatite saturation temperatures using bulk rock compositions of granites characterized by inheritance-rich and -poor zircon populations originally compiled by Miller et al. (2003). Apatite saturation temperatures calculated with Eq. 6, zircon saturation temperatures calculated using calibration of Watson and Harrison (1983). Histograms on right and top axes respectively show the distribution of calculated zircon and apatite saturation temperatures for the two granite types

Finally, to highlight the complexities present in applying apatite saturation thermometry in granitic rocks we compare zircon and apatite saturation temperatures calculated for the granitic dataset compiled by Miller et al. (2003). This study found systematic differences in zircon saturation temperatures between samples with inheritance-rich and inheritance-poor or -free zircon populations. These two populations were originally interpreted to represent initial emplacement of ‘cold’, crystal-rich magmas and ‘hot’, crystal-poor magmas, respectively. As observed in the original publication, the inheritance-rich granites have uniformly lower Zr contents than the inheritance-poor granites, indicating that the inherited zircon cores do not dominate the Zr budget of these samples (Miller et al. 2003). However, calculated apparent apatite saturation temperatures do not reproduce the same systemics (Fig. 11; note that for the remainder of this case study, unless specified otherwise, all saturation temperatures should be considered *apparent* saturation temperatures but for readability we drop this phrasing). Apatite saturation temperatures are generally higher than zircon saturation temperatures, and there is significant scatter in apatite saturation temperatures in both the zircon inheritance-rich and -poor populations. Further, the difference in average apatite saturation temperature of the zircon inheritance-rich granite population is only marginally lower than that of the zircon inheritance-poor granites (862 vs. 890 °C).

The lack of coherency between the zircon and apatite saturation temperatures is to be expected. These two thermometers should only agree when the granitoid source was initially saturated in both phases, most plausibly occurring when the granitic liquid was extracted from a mushy magma containing melt in equilibrium with apatite and zircon (as well as other major phases). In this case, the two thermometers should agree and would constrain the temperature of melt extraction from this mushy reservoir, or equivalently of melt emplacement at the plutonic level. This may be the case for the small number of samples where the two thermometers agree within uncertainty, and within that subset seems particularly likely for the two zircon inheritance-rich granitoids that agree within uncertainty.

The remainder of the samples are characterized by elevated apatite saturation temperatures relative to zircon saturation temperatures. Systematically elevated apatite saturation temperatures relative to zircon saturation temperatures have been described in previous studies, where they were interpreted as evidence of early (near-liquidus) apatite saturation (e.g., Anderson et al. 2008), or occasionally attributed to difficulty with existing apatite saturation thermometers in modeling peraluminous compositions (e.g., Hoskin et al. 2000). As our apatite saturation thermometer performs well for typical peraluminous liquids (Fig. 9a), systematic model biases in peraluminous granitoids as a potential cause of this offset are unlikely. Instead, we envision two potential scenarios that explain the observed apatite saturation systematics: (1) similar to prior interpretations, elevated apatite saturation temperatures may result when apatite was a (near-)liquidus phase in an equilibrium or closed fractionation system, or at least when it saturates earlier than zircon. If apatite is a liquidus phase, apatite saturation temperatures approximate the liquidus or equivalently the temperature of melt extraction from the prior mushy magma system already in equilibrium with apatite. By contrast, if the system is not initially saturated in either zircon or apatite, the relationship between the two thermometers will be more complex, but higher (apparent) apatite saturation temperatures would still likely indicate that apatite saturated prior to zircon. (2) Alternatively (or additionally), elevated apatite saturation temperatures may be reflective of the selected granitoids not approximating liquid compositions. This could result from accumulation of low-SiO_2_ major phases (plagioclase, biotite, amphibole) resulting in lower bulk rock SiO_2_ which would yield higher saturation temperatures, or from either in situ accumulation, or antecrystic, inherited or xenocrystic apatite resulting in elevated bulk rock P_2_O_5_ contents.

The presence or absence of zircon inheritance may help to distinguish between these two processes. In particular, the presence of inherited zircon cores in the inheritance-rich samples provides strong evidence that these melts were zircon-saturated at emplacement, in which case the zircon saturation temperatures should approximate the emplacement temperature (again, this requires that the volume of inherited zircon is small and that other accumulation processes were not significant). Given the low zircon saturation temperatures calculated for these samples (~ 750–800 °C), we suspect that most of these samples were also saturated in apatite at emplacement, and for closed-system crystallization we would then expect the apatite saturation temperatures to also approximate the emplacement temperatures. Instead, the apatite saturation temperatures are significantly higher for most samples, which we suggest is most likely due to accumulation of low-SiO_2_ major phases and/or apatite.

While the importance of crystal accumulation in granitoid formation is relatively well-established, the potential for significant apatite accumulation and/or inheritance is intriguing, particularly given that comparatively few instances are documented in the literature (c.f., Clarke et al. 2023; Laurent et al. 2017; Sun et al. 2021; Tepper and Kuehner 1999; Zhan et al. 2022). We suspect that the limited number of examples may, in part, reflect the fact that relative to zircon different types of apatite inheritance are typically far less conspicuous. Low closure temperatures in the U–Pb system in apatite (e.g., Cochrane et al. 2014) mean that non-autocrystic apatite will almost certainly not preserve primary geochronologic information in slowly cooled intrusive rocks. Similarly, high F and H_2_O diffusivities (Li et al. 2020) make it likely that these species will equilibrate with the entraining melt and inherited apatites will typically not preserve original volatile compositions. Instead, future efforts to identify non-autocrystic apatite should focus on careful back scattered electron and cathodoluminescence imaging and analyses of slower diffusing species including sulfur, CO_2_, Mn and rare earth elements (Cherniak 2000, 2005; Li et al. 2020).

## Conclusions

This study presents a new model for apatite saturation based on a compilation of apatite-bearing experimental melt compositions supplemented with data from natural rhyolitic glasses. The model is presented as two separate equations optimized to predict melt temperatures and P_2_O_5_ contents respectively. Similar to most previous accessory mineral saturation models, the model is based on an Arrhenius-type relationship represented as a linear correlation between the logarithm of phosphorus contents and inverse temperature, with additional sensitivity to melt compositional parameters: SiO_2_ contents and aluminum saturation index (ASI). The Arrhenius relationship results in a thermometer form that performs well, with typical errors < 35 °C across a wide range of melt compositions ranging from basalts to rhyolites, including alkaline and most peraluminous compositions, and is robust to even modest analytical errors in phosphorus measurements. In contrast, this relationship results in a model for P_2_O_5_ contents as a function of temperature that is somewhat less precise when considering absolute phosphorus contents. We find that at crustal conditions apatite saturation is insensitive to crystallization pressure, and as long as an appropriate volatile anion species is present (OH-, F-, Cl-), is also insensitive within model resolution to volatile abundances. This new model significantly outperforms existing apatite saturation models and is accurate for both metaluminous and peraluminous melts, as illustrated for multiple case studies. However, it does not accurately model apatite saturation in the subset of peraluminous melts that are highly phosphorus enriched. These melts can be identified based on having molar CaO:P_2_O_5_ ratios lower than stoichiometric apatite. Apatite saturation in these liquids is likely much more strongly controlled by CaO contents.

## Supplementary Information

Below is the link to the electronic supplementary material.

Supplementary file1 (PDF 497 KB)

Supplementary file2 (XLSX 190 KB)

## Data Availability

All new and compiled dataused in this study are included in the supplementary materials.
