# Supplementary material for: Apatite saturation revisited: new model formulations and applications to igneous rocks
Source: Contrib Mineral Petrol. 2026 Feb 19;181(3):18. doi: 10.1007/s00410-026-02300-5 (PMC12920737; doi:10.1007/s00410-026-02300-5)
Supplement: Supplementary file 1 — Supplementary file1 (PDF 497 KB) [file 410_2026_2300_MOESM1_ESM.pdf]

Supporting information for

Apatite Saturation Revisited: new model formulations and applications to igneous rocks

Benjamin Z. Klein[1], Othmar Müntener[1], Jack Gillespie[1], Felix Marxer[2]

[1] Institute of Earth Sceinces, University of Lausanne, Switzerland; [2] Institute of Earth System Sciences, Leibniz University Hannover, Germany

Contents of this file

Text S1: Detailed description of analytical methods
Figures S1 to S2: Supplemental figures
Table S1 (uploaded as separate excel file): Compilation of apatite-saturated experiments

Detailed analytical methods

Electron Probe Microanalyzer (EPMA)

Analyses of the phosphorus contents of previously published apatite-bearing experimental liquids in Marxer and Ulmer (2019) were made using the JEOL JXA-8530F field emission gun electron probe microanalyzer (EPMA) housed in the Institute of Earth Sciences at the University of Lausanne. Analyses were performed using a 15 kV accelerating voltage and a 10 nA beam current. Analyses of experiments with larger melt pockets were conducted with a defocused electron beam with a 10-20 μm diameter, but smaller diameters (3-5 μm diameter) were employed when required due to small melt pockets. Phosphorus was measured using 60 or 90 second on-peak counting times and 30 or 45 second off-peak background measurements, while all other major elements were measured for 30 seconds on-peak and 15 seconds off-peak, except for Na and K which were each measured for 10 seconds on-peak and 5 seconds off peak. Calibration standards used for analysed elements included albite (Si, Na), rutile (Ti), orthoclase (Al, K), fayalite (Fe), wollastonite (Ca), rhodonite (Mn), forsterite (Mg), and apatite (P). Raw data were corrected for matrix effects using the CITZAF package (Armstrong 1995). Major element analyses of most glasses agree closely with the original data published in Marxer and Ulmer (2019). However, the new measurements were made with higher beam currents to optimize the phosphorus measurements which, particularly for the smaller beam diameter analyses, resulted in beam damage to the glasses and less accurate alkali measurements. Therefore, in the calibration dataset we use the original published analyses for major elements combined with the newly analyzed P_2O_5 contents.

Laser ablation inductively coupled plasma mass spectrometry (LA-ICP-MS)

Measurements were performed on the four apatite-bearing experiments originally published in Nandedkar et al., (2014). Measurement were made using an Australian Scientific Instrument RESOlution 193 nm Ar-F excimer laser with an S155 dual volume sample cell, interfaced to a sector field Element XR ICP-MS mass spectrometer (Thermo Scientific) at the University of Lausanne. The LA-ICP-MS system was optimized by linearly scanning the NIST SRM-610 glass standard at 10 μm s^{-1} to increase the spectrometer sensitivity ($^{139}La^+$ >2.5 × 10^6 cps) without significantly producing oxides ($^{248}ThO^+/^{232}Th^+$ <0.2 %) and doubly-charged ions (Ba^{2+}/Ba^+ <2.5 %). At least 10 measurements on large glass pools were acquired for each experiment. Replicative measurements were done with a rectangular laser pit shape of 12.5 by 37.5 μm using a frequency of 10 Hz and an energy density of ~6 J cm^{-2}. Helium (500 ml min^{-1}) was used as a carrier gas. Background, ablation

and wash-out interval acquisition times on primary and secondary standards were 70 s, 10.3 s and 35 s, respectively. Ablation interval acquisition times on hydrous glasses varied between 2.5 - 10.7 s depending on the complexity of the signal (e.g. mineral inclusions). Dwell times range from 10 to 20 ms depending on the measured isotope. Absolute concentrations were calculated using Al_2O_3 (measured by EPMA, Nandedkar et al. 2014) as internal reference, NIST SRM-610 as primary standard, and BCR-2G as secondary standard for quality control. Data reduction was performed using the software LAMtrace (Jackson 2008). The average elemental abundances of the standards were taken from Pearce *et al.* (1997). The updated LA-ICP-MS data for these experiments are reported in Supplementary Table 1. The new P_2O_5 LA-ICP-MS measurements agree within uncertainty with the originally published EPMA analyses (Supplementary Figure S2).

Additional References

Armstrong JT (1995) Citzaf-a package of correction programs for the quantitative Electron Microbeam X-Ray-Analysis of thick polished materials, thin-films, and particles. Microbeam Analysis 4:177–200

Jackson SE (2008) LAMTRACE data reduction software for LA-ICP-MS. In: Sylvester P (ed) Laser Ablation ICP-MS in the Earth Sciences: Current Practices and Outstanding Issues. Mineralogical Association of Canada Short Course Series, 40th edition.

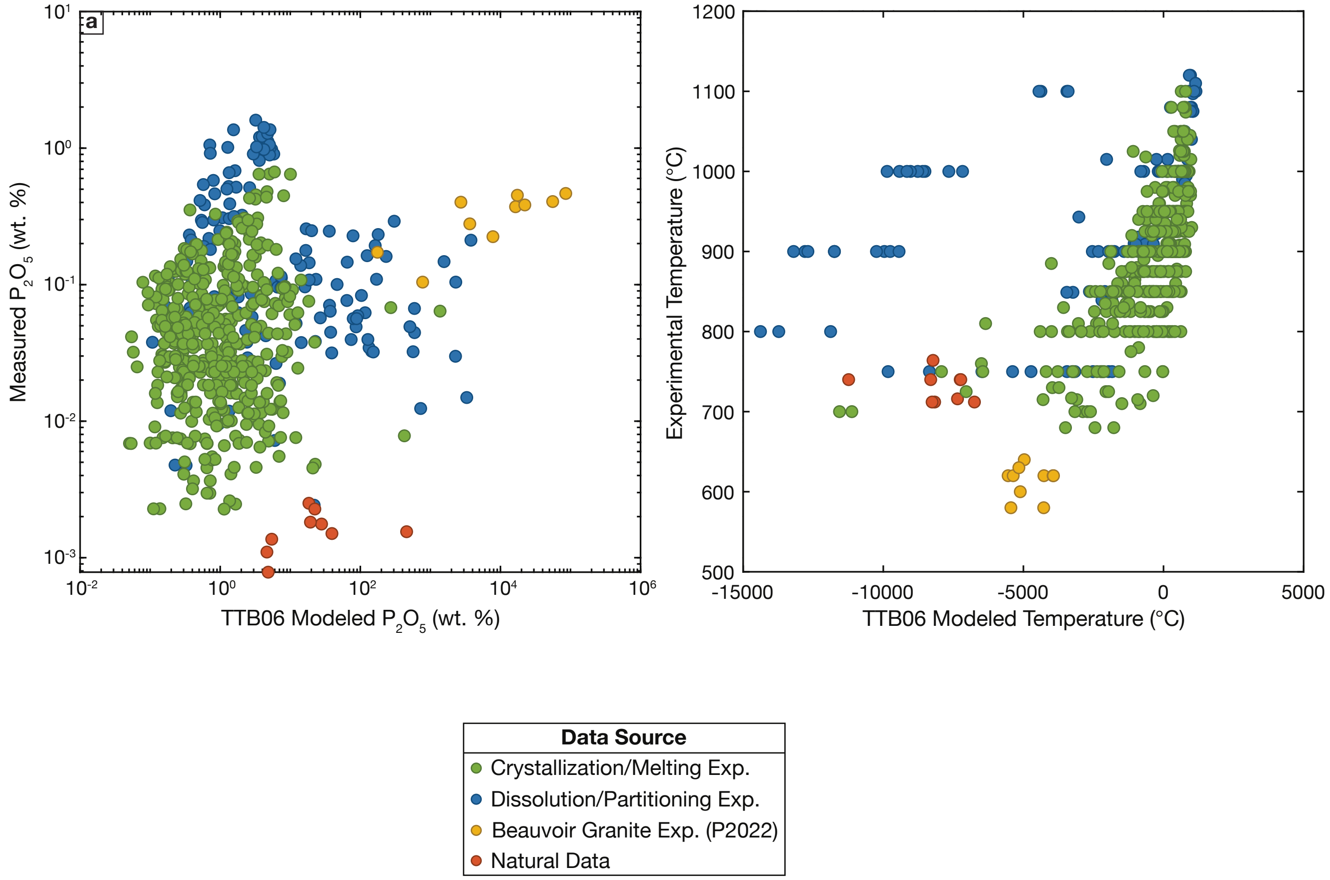

Supplementary Figure 1. Evaluation of temperatures (a) and phosphorus contents (b) calculated using the model from Tollari et al., (2006) compared to experimental data. Only data that satisfy the filtering criteria discussed in the main text are plotted. Symbology as in main text Figure 1.

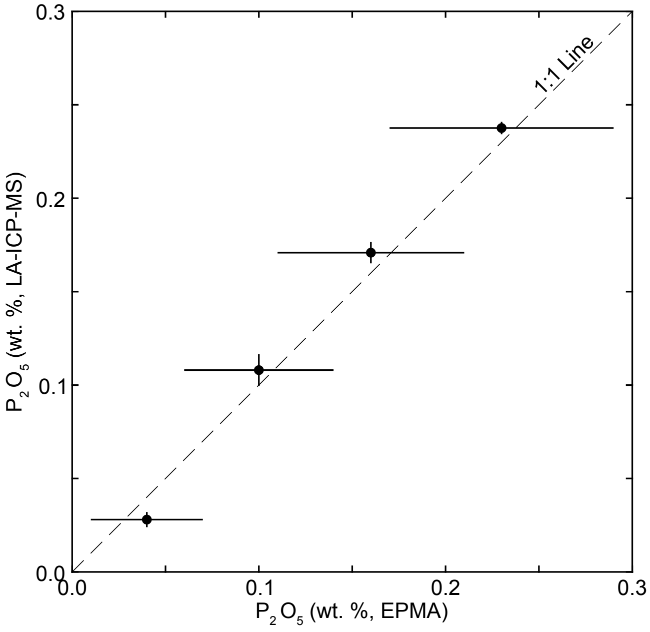

Supplementary Figure 2. Comparison of EPMA and LA-ICP-MS measured P_2O_5 contents of glasses in apatite-bearing experiments from Nandedkar et al. (2014).
